# Estimation of human age using *N*-glycan profiles from bloodstains

**DOI:** 10.1007/s00414-015-1162-x

**Published:** 2015-03-19

**Authors:** Ivan Gudelj, Toma Keser, Frano Vučković, Vedrana Škaro, Sandra Šupraha Goreta, Tamara Pavić, Jerka Dumić, Dragan Primorac, Gordan Lauc, Olga Gornik

**Affiliations:** 1 Genos Ltd., Glycobiology Department, Hondlova 2, 10 000 Zagreb, Croatia; 2 Faculty of Pharmacy and Biochemistry, University of Zagreb, A. Kovačića 1, 10 000 Zagreb, Croatia; 3 School of Medicine, University of Osijek, Josipa Huttlera 4, 31 000 Osijek, Croatia; 4 Eberly College of Science, Penn State University, University Park, Penn, PA USA; 5 School of Medicine, University of Split, Šoltanska 2, 21 000 Split, Croatia; 6 St. Catherine Hospital, Bračak 8, Zabok, Croatia

**Keywords:** Bloodstain, N-glycosylation, Aging, Age estimation

## Abstract

Protein glycosylation is the most common epiproteomic modification involved in numerous physiological and pathological processes. Previous studies reported strong associations between human plasma *N*-glycans and age, prompting us to evaluate the potential application of this biological phenomenon in the field of forensics. Blood from 526 blood donors from different parts of Croatia was collected on bloodstain cards during the period 2004–2007 and stored at 4°C for 6–9 years. Glycosylation profiles of the bloodstains were analysed using hydrophilic interaction ultra performance liquid chromatography (HILIC-UPLC) and divided into 38 glycan groups (GP1-GP38). A statistically significant correlation between *N*-glycan profiles of bloodstains and chronological age was found and a statistical model that can be used for the age prediction was designed (Age = 75.59 – 5.15 × (GP4)^2^+ 17.07 × GP6 – 5.30 × (GP10)^2^ – 16.56 × GP16 + 20.07 × GP20 – 7.54 × (GP20)^2^ + 16.47 × GP22). This model explains 47.78 % of the variation in age, with a prediction error of 9.07 years. Our findings demonstrate that analysing the *N*-glycan profile could be a new tool in forensics, offering an approximate human age estimation from dried bloodstains found at a crime scene.

## Introduction

Assessing the age of a living person is a very important task in forensics. Until now, age estimation depended mostly on morphological methods, but forensic samples, such as bloodstains, do not contain this information. Researchers have tried different approaches to estimate human age from a blood sample, such as using the mitochondrial deletion frequency in human blood and different types of blood cells [1], but all tested approaches still show limitations. On the other hand, telomere-based age analysis requires a relatively large amount of intact DNA that is not always available in forensic cases [1]. Additionally, the DNA is often degraded, showing a shorter terminal restriction fragment (TRF) length, even in cases when dried bloodstains were stored for five months and then processed.

Protein glycosylation is the most common post-translational modification of proteins. This process is not random and is controlled by enzymatic addition of sugars to proteins. Our recent population studies showed significant variations in glycome composition between individuals [2, 3]. Glycan chains attached to protein backbones are involved in nearly all molecular interactions on the cell surface and in the intracellular space. Changes in levels and composition of glycans were reported as being associated with numerous physiological features, such as age, gender, race, reproductive cycle, developmental stage, environmental influences, body mass index, plasma lipid status, and dietary and lifestyle habits [4]. Despite these, the plasma glycan profile of a single individual is under rather strong genetic influence [5] and is remarkably stable over a short period of time [6]. It has been known for over 20 years that changes in human plasma *N*-glycans correlate with age [7] and that this correlation, statistically significant for many glycan structures, is higher in females than in males [8]. We have also come upon similar findings for immunoglobulin G glycans, which explain 58 % of the variation in chronological age [9, 10], and from which a person’s age can be estimated with an error of 9.7 years.

In this study, we investigated the association between *N*-glycan profiles acquired from dried bloodstains of 526 individuals and their age, aiming to evaluate potential forensic application of this analysis. We used dried blood samples that were stored on absorbent filter paper for 6 to 9 years. Dried blood spot sampling is a very simple technique for collecting, storing and shipping blood samples and is widely used in applications such as screening for metabolic and sickle cell disorders, as well as for HIV and malarial infections. This method involves collection of 15 μL of blood on absorbent filter paper that can be subsequently shipped and stored between –20°C and tropical temperatures [10]. Our previous studies demonstrated a very strong association of IgG glycans with age and here we expanded this study to N-glycans obtained from bloodstains, a sample that may be relevant for forensics.

## Materials and methods

### Samples

Blood samples were collected from 526 blood donors from different parts of Croatia (Bjelovar, Dubrovnik, Osijek, Pula, Sisak, Split, Šibenik, Zabok, Zagreb). Of the 526 participants 402 were men (age 35 (18–63)) and 124 were women (age 29 (18–77)). Bloodstains were collected during volunteer blood donation. Capillary blood was collected on Whatman Schleicher & Schuell bloodstain cards from 2004 to 2007 and stored at +4°C. Sample collection was conducted according to the Declaration of Helsinki Ethical Principles for Medical Research Involving Human Subjects and was approved by the ethics committees of the University Hospital Centre Zagreb and the Faculty of Pharmacy and Biochemistry at the University of Zagreb.

### Condition testing

Blood was drawn from one individual and put on Whatman Schleicher & Schuell bloodstain cards and a kitchen cloth. Bloodstains from bloodstain cards were subsequently exposed to different conditions (humidity, different temperatures and UV radiation). Humidity testing was conducted in a water incubation chamber at +37°C for 6 days. The influence of different temperatures was determined by exposing the bloodstains to either +4 °C in the fridge or +37°C and +65°C in the oven for 6 days, while the influence of UV radiation was measured by placing bloodstains under a UV lamp (60 W, 254 nm) for 20 min or leaving them in a room with a UV lamp (15 W, 253.7 nm) for 2 h. Bloodstains on the kitchen cloth were stored at room conditions, and bloodstain cards with blood were used as reference samples. Each experiment was done in tetraplicate.

### Glycan release and labeling

From each bloodstain card, a circle with a diameter ≈ 6 mm was cut and transferred onto a microtiter plate. Each following step was done in a 96-well microtiter plate to achieve the best throughput of sample preparation. After adding 10 μL of ultra-pure water into each well with a sample of blood stain cards, samples were first denatured by addition of 20 μL of 2 % sodium dodecyl sulfate (SDS; w/v; Invitrogen, Carlsbad, CA, USA) and by incubation at 65 °C for 10 min. Subsequently, 10 μL of 4 % Igepal-CA630 (Sigma-Aldrich, St. Louis, MO, USA) and 1.25 mU of PNGase F (ProZyme, Hayward, CA, USA) in 10 μL of 5× phosphate-buffered saline (PBS) were added to the samples. The samples were incubated overnight at 37°C for *N*-glycan release. The released *N*-glycans were labeled with 2-aminobenzamide (2-AB). The labeling mixture was freshly prepared by dissolving 2-AB (Sigma-Aldrich, St. Louis, MO, USA) in a dimethyl sulfoxide (DMSO; Sigma-Aldrich, St. Louis, MO, USA) and glacial acetic acid (Merck, Darmstadt, Germany) mixture (85:15, v/v) to a final concentration of 48 mg/mL. A volume of 25 μL of labeling mixture was added to each *N*-glycan sample in the 96-well plate. Also, 25 μL of freshly prepared reducing agent solution [106.96 mg/ml 2-picoline borane (Sigma-Aldrich, St. Louis, MO, USA) in DMSO] was added and the plate was sealed using adhesive tape. Mixing was achieved by shaking for 10 min, followed by 2 h incubation at 65°C. The liquid component of the samples (approximately 100 μL, without paper) was transferred into a new 96-well plate and was brought to 80 % acetonitrile (ACN; v/v) by adding 400 μL of ACN (J.T. Baker, Phillipsburg, NJ, USA). Free labeling and reducing agents were removed from the samples using microcrystalline cellulose. An amount of 200 μL of 0.1-g/mL suspension of microcrystalline cellulose (Merck, Darmstadt, Germany) in water was applied to each well of a 0.45-μm GHP filter plate (Pall Corporation, Ann Arbor, MI, USA). Solvent was removed using a vacuum manifold (Millipore Corporation, Billerica, MA, USA). All wells were prewashed five times using 200 μL of water, followed by equilibration using three washes of 200 μL of acetonitrile/water (80:20, v/v). The samples were loaded in the wells of the GHP filter plate and the wells were subsequently washed seven times using 200 μL of acetonitrile/water (80:20, v/v). Glycans were eluted twice with 100 μL of water and combined eluates were stored at −20°C until usage.

### Hydrophilic interaction chromatography (HILIC)-UPLC

Fluorescently labeled *N*-glycans were separated by hydrophilic interaction chromatography on a Waters Acquity UPLC instrument (Milford, MA, USA) consisting of a quaternary solvent manager, sample manager and a FLR fluorescence detector set with excitation and emission wavelengths of 330 and 420 nm, respectively. The instrument was under the control of Empower 2 software, build 2145 (Waters, Milford, MA, USA). Labeled *N*-glycans were separated on a Waters BEH Glycan chromatography column (150 × 2.1 mm i.d., 1.7-μm BEH particles, with 100-mM ammonium formate, pH 4.4, as solvent A and acetonitrile as solvent B). The separation method used a linear gradient of 75–53 % acetonitrile (v/v) at a flow rate of 0.56 ml/min in a 25-min analytical run. Samples were maintained at +5 °C before injection, and the separation temperature was +25 °C. The chromatograms were all separated in the same manner into 38 chromatographic regions that enabled reliable quantification. The amount of glycans in each peak was expressed as percentage of total integrated area.

### Statistical analysis

To obtain normally distributed variables for 38 glycan structures, a log transformation was performed on the glycan variables. The predictive model of chronological age was built using a multivariate regression approach implemented in the “stats” package for the R programming language. The maximal model included linear and quadratic terms for each of the 38 glycan peaks, giving 76 parameters in total. Feature selection was performed using a backward elimination procedure with Akaike information criterion used as optimization criteria. Reported goodness of fit (coefficient of determination), prediction errors and regression coefficients were estimated as median values of 100 iterations of repeated, random sub-sampling validation, with two thirds (347 samples) of the sample set as a “training set” and one third (174 samples) as a "validation set."

## Results

**Fig. 1:**
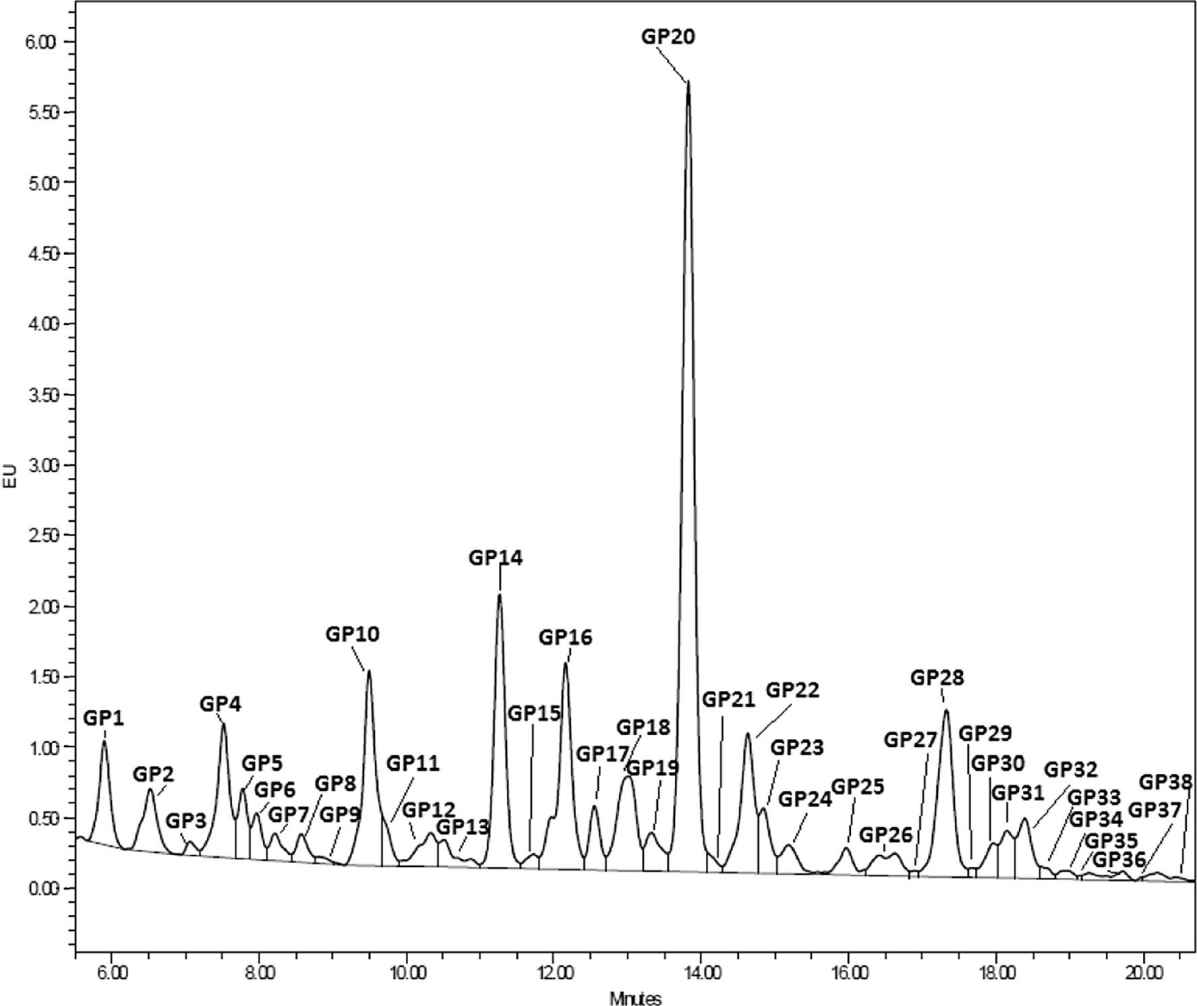
HILIC-UPLC chromatogram of the bloodstain N-glycome. The chromatogram was separated into 38 peaks

After HILIC-UPLC analysis of the *N*-glycans released from the 526 bloodstain samples, the chromatograms were divided into 38 glycan groups (Fig. 1), each containing different glycan structures.

In order to search for the connection between glycans and age, a maximal prediction model, which included linear and quadratic terms of glycan groups, was created (not shown).

To obtain a more accurate and simple age prediction model, the number of parameters was further reduced using a backward elimination procedure, and, after the features were selected, a final prediction model containing six glycan groups was derived.

$$ Age=75.59-5.15\times {(GP4)}^2+17.07\times GP6-5.30\times {(GP10)}^2-16.56\times GP16+20.07\times GP20-7.54{(GP20)}^2+16.47\times GP22 $$

**Fig. 2:**
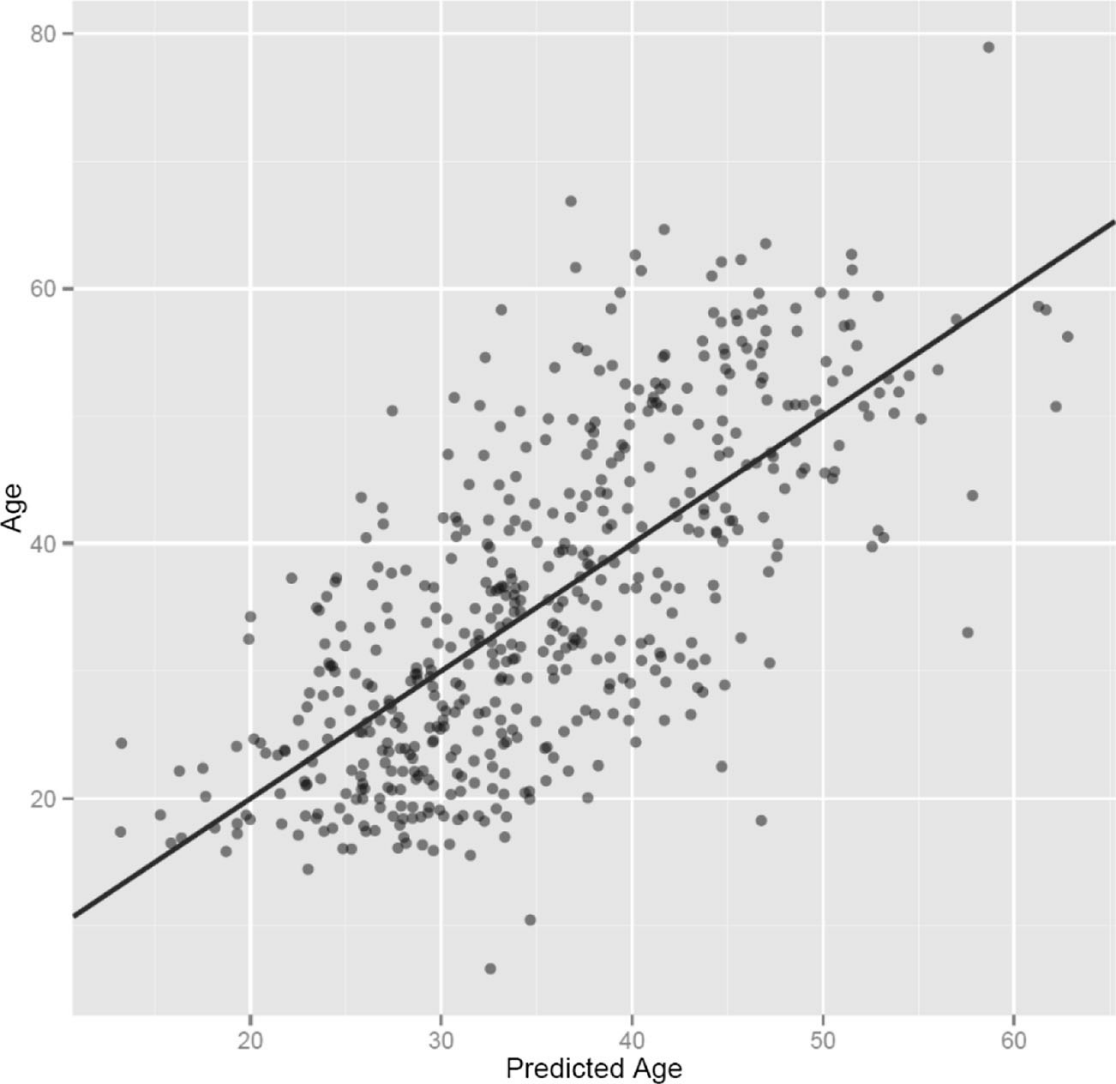
Correlation of the age predicted by the model designed in this study and the real chronological age. Predicted age was calculated for 526 individuals from the N-glycan profiles of their bloodstains. The model is slightly better at determining a woman’s age than a man’s (55.97 % vs. 44.28 %)

The formula includes six glycan peaks (GP4, GP6, GP10, GP16, GP20 and GP22) and can explain 47.78 % of the age variation and predict a person’s age with an error of 9.07 years. It describes age for women slightly better than for men (55.97 % vs. 44.28 %), comfirming previous findings [4]. Correlation of the age predicted by the model and the real chronological age is shown in Fig. 2.

**Fig. 3:**
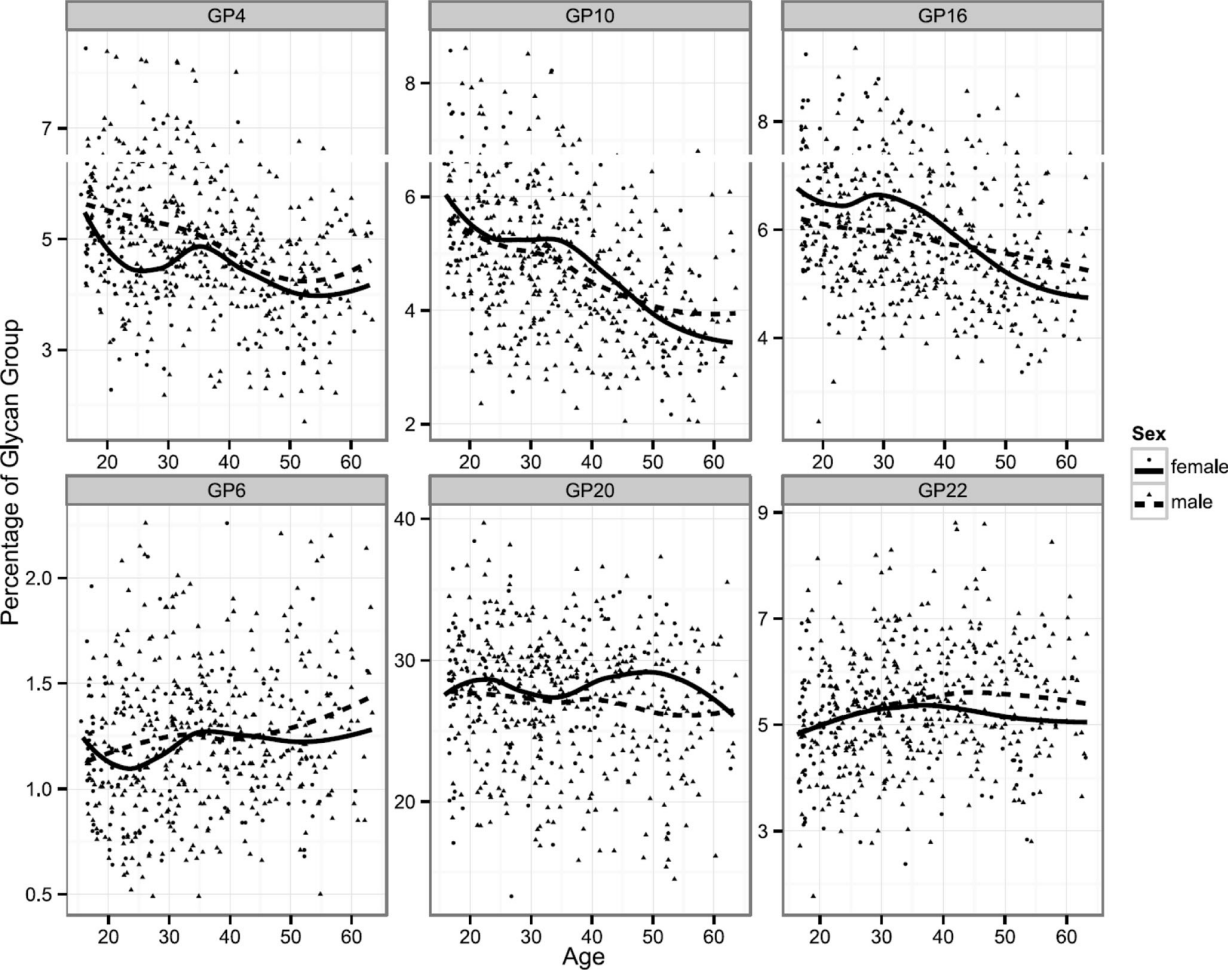
Relationship between age and glycan groups included in the final age prediction model. Plots indicate associations between the individual contributions of six glycan groups to the total dried bloodstain glycomes and chronological age of participants. Curves are fitted local regression models describing gender-specific relationship between age and glycan group

An association with age for each glycan group included in the age prediction model can be seen in Fig. 3, while their individual influence on the model and the coefficient of their correlation with age were determined by statistical analysis and are given in Table 1. The analytical precision of the method was calculated from 11 repeated measurements of peaks included in the age estimation model and is given in Table 2. From the table it can be seen that their average coefficient of variation (CV) is 14.53 %.

**Table 1:** Association of Dried Blood Spot Glycans With Age

Glycan group	R	p
GP1	0.18	4.72E-05
GP2	0.22	3.15E-07
GP3	0.10	1.75E-02
GP4	−0.25	3.48E-09
GP5	−0.09	2.98E-02
GP6	0.16	1.63E-04
GP7	−0.02	6.50E-01
GP8	−0.10	1.67E-02
GP9	0.04	3.36E-01
GP10	−0.35	9.27E-17
GP11	0.00	9.71E-01
GP12	0.15	5.24E-04
GP13	0.10	1.87E-02
GP14	0.03	5.40E-01
GP15	0.08	5.93E-02
GP16	−0.21	7.01E-07
GP17	0.06	2.05E-01
GP18	−0.02	5.95E-01
GP19	0.05	2.58E-01
GP20	0.00	9.80E-01
GP21	0.10	1.65E-02
GP22	0.14	1.46E-03
GP23	0.18	3.34E-05
GP24	0.08	5.25E-02
GP25	0.07	1.08E-01
GP26	0.20	3.61E-06
GP27	0.08	7.02E-02
GP28	0.01	8.49E-01
GP29	0.22	3.99E-07
GP30	0.17	5.60E-05
GP31	0.10	1.61E-02
GP32	0.12	6.09E-03
GP33	0.30	1.56E-12
GP34	0.26	2.15E-09
GP35	0.18	4.07E-05
GP36	0.11	9.08E-03
GP37	0.10	1.70E-02
GP38	0.16	3.04E-04

*GP* glycan peak, abbreviation usually used to denote the group of glycans that co-elute in chromatography, since chromatographic peaks may sometimes contain more than one structure; *R* coefficient of correlation

**Table 2:** The analytical precision of the method given as coefficients of variation for glycan groups included in the age estimation formula. The CV is calculated from 11 repeated measurements of the same sample

Glycan group	GP4	GP6	GP10	GP16	GP20	GP22	average
CV	11.19	22.14	14.6	10.69	16.54	12.0	14.53

*GP* glycan peak, abbreviation usually used to denote the group of glycans that co-elute in chromatography, since chromatographic peaks may sometimes contain more than one structure; *CV* coefficient of variation

The influence of different environmental factors, to which bloodstains were exposed (see Methods section), on age estimation is given in Table 3. Plasma glycans were analyzed and the age was calculated using the proposed formula.

**Table 3:** Influence of different environmental conditions on age estimation from bloodspots. Estimated age is given as a median (range)

Condition	RC	UV 20 min	UV 2 h	4°C	37°C	65°C	humidity	cloth
age (years)	53 (52–55)	54 (54–56)	56 (54–59)	54 (53–55)	53 (52–55)	52 (50–53)	50 (48–55)	50 (49–54)

*RC* room conditions; *UV* ultra violet; *humidity* blood spot exposed to humid environment; *cloth* blood spot put on kitchen cloth

## Discussion

Estimation of human age from the biological samples represents an important tool in forensic medicine and crime scene investigation, but the precise methods for age prediction, which include samples with limited morphological or biochemical information, are still lacking. Previous studies showed a good correlation between the serum *N*-glycome and aging, indicating the potential of glycans as biomarkers of age [9, 11]. To evaluate this potential in forensic samples, we analysed glycans from 526 bloodstains collected 6–9 years prior to this study. After releasing and labeling of the glycans, HILIC–UPLC chromatography enabled us to separate the bloodstain glycome into 38 chromatographic peaks and to design the formula by which a person’s age can be estimated. This formula includes six glycan peaks and can explain 47.78 % of the age variation and predict a person’s age with an error of 9.07 years. The formula determines age estimation for women slightly better than men (55.97 % vs. 44.28 %), which coincides with previous findings [4].

When comparing the age estimation using N-glycans obtained from bloodstains to the one using IgG *N*-glycome, the bloodstains method displayed slightly weaker results (IgG *N*-glycome coefficient of determination R^2^ = 58.0 %). Despite that, the bloodstain method shows other advantages, as it requires a very small amount of sample and is more suitable for crime scene conditions than the IgG method, which requires a relatively large amount of sample (50 – 90 μL of plasma) and includes the IgG purification step.

Recent studies tested a similar approach to age estimation using bloodstains. One of those methods was based on telomere shortening. A majority of publications that tried to associate telomere length with age showed results that were inadequate for forensic application (e.g., R^2^ = 0.04 [11] or 0.05 [12]). One study done by Tsuji A. et al. showed a coefficient of determination of R^2^ = 69.2 % [13], but, still, this method has some disadvantages, such as DNA degradation. If the DNA is cut to a size smaller than 500 bp this method is not applicable. Also, this result is valid only for fresh blood samples, since it was shown that dried bloodstains stored for 5 months had a mean TRF length 500 bp shorter than the ones from freshly drawn blood samples. Beside this, in this study, a Southern blotting method was used which requires a relatively large amount of DNA, which is not always available at a crime scene. It is also important to mention that this study was done on only 60 Japanese individuals and the influence of the population features, due to different environmental and lifestyle factors, must not be neglected.

The second method, which used a similar approach, is based on predicting human age by signal joint T-cell receptor rearrangement excision circle (sjTREC) quantification. It also showed a higher coefficient of determination compared to our method (R^2^ = 76 %), but it should have taken into consideration the fact that sjTREC level in blood can be changed by numerous factors, since it is a function of thymic output. Thus this method requires studying the potential impact of genetic and disease factors. The loss of 0.16 - 1.93 dCt was detected after 1.5 years of storage, and the results after storing for 3, 6, 12 and over 20 years in laboratory conditions showed a time-dependent decrease in the correlation coefficient R, even though the sjTREC contents were all detectable in these old bloodstain samples [14].

The level of glycosylation of human plasma proteins is under strong genetic regulation, with heritability of over 80 % for certain glycans [2, 3]. However, environmental influences [4], as well as the influence of different diseases [15] and lifestyle [4] also exist for this feature. Nowadays, a lot of effort is being put into discovering the level of this influence. One should be aware of the significant scattering of data shown in this paper, which is probably the consequence of the high population variability of glycans [2] and aforementioned influences. Nevertheless, our results show that a bloodstain could be used to approximate the age of an offender who committed a crime, even after a few years have past. This represents a valuable advancement in the field of forensics. Moreover, intraindividual stability of the plasma N-glycan profile was shown [6], and when comparing this method to methods mentioned above, it requires a relatively small amount of sample (less than 5 μL of blood), which usually corresponds to a crime scene situation. Even under extreme conditions (high humidity, temperature and UV radiation) in the case when bloodstains were collected from the cloth, which is more similar to a real sample from a crime scene, the method showed age estimation with an acceptable error. This error was comparable to the analytical performance of the method.

Until recently, the analysis of glycan structures represented an impossible mission, due to their structural diversity and lack of adequate analytical methods. In the last few years, this field has experienced great development [16] and further improvements could also enable advances in usage of glycans for the purpose tested in this paper. The improvement in critical steps of glycan analysis, such as purification and labeling, should also ameliorate the value of glycan usage in the field of forensics. Moreover, the combination of glycans with other features used for age estimation could probably yield better results.

A recent comprehensive report by the US National Academy of Science identified the great underexplored potential of glycans in numerous diseases [14] and, apparently, this should also be expanded to the field of forensics.

Even so, none of the methods mentioned above is satisfactory for estimating human age from a sample that contains no morphological or biochemical information, yet the combination of these methods could be useful for accurate age prediction.
